# Using areas of known occupancy to identify sources of variation in detection probability of raptors: taking time lowers replication effort for surveys

**DOI:** 10.1098/rsos.160368

**Published:** 2016-10-12

**Authors:** Campbell Murn, Graham J. Holloway

**Affiliations:** 1 Hawk Conservancy Trust, Andover, Hampshire SP11 8DY, UK; 2 School of Biological Sciences, University of Reading, Berkshire RG6 6AS, UK

**Keywords:** detection probability, raptors, vultures, *Trigonoceps occipitalis*, absence, presence

## Abstract

Species occurring at low density can be difficult to detect and if not properly accounted for, imperfect detection will lead to inaccurate estimates of occupancy. Understanding sources of variation in detection probability and how they can be managed is a key part of monitoring. We used sightings data of a low-density and elusive raptor (white-headed vulture *Trigonoceps occipitalis*) in areas of known occupancy (breeding territories) in a likelihood-based modelling approach to calculate detection probability and the factors affecting it. Because occupancy was known *a priori* to be 100%, we fixed the model occupancy parameter to 1.0 and focused on identifying sources of variation in detection probability. Using detection histories from 359 territory visits, we assessed nine covariates in 29 candidate models. The model with the highest support indicated that observer speed during a survey, combined with temporal covariates such as time of year and length of time within a territory, had the highest influence on the detection probability. Averaged detection probability was 0.207 (s.e. 0.033) and based on this the mean number of visits required to determine within 95% confidence that white-headed vultures are absent from a breeding area is 13 (95% CI: 9–20). Topographical and habitat covariates contributed little to the best models and had little effect on detection probability. We highlight that low detection probabilities of some species means that emphasizing habitat covariates could lead to spurious results in occupancy models that do not also incorporate temporal components. While variation in detection probability is complex and influenced by effects at both temporal and spatial scales, temporal covariates can and should be controlled as part of robust survey methods. Our results emphasize the importance of accounting for detection probability in occupancy studies, particularly during presence/absence studies for species such as raptors that are widespread and occur at low densities.

## 1. Introduction

Detection probability and the consequences of imperfect detection (i.e. false absences) during species monitoring or occupancy assessments has been a consideration of importance to fieldworkers and analysts for some time [1,2], as has the assessment and analysis of detection probability [3–5]. Where imperfect detection does occur or is not accounted for, models used to estimate occupancy will be inaccurate and broader measures of populations, community compositions and species distribution models will probably be biased [6]. A further problem with imperfect detection is that although monitoring programmes strive to maintain consistent methods and effort (i.e. be standardized), the probability of detecting an elusive species is not constant in space or time. In reality, detection probability is rarely if ever constant, either between survey sites or between individual surveys [7], because it is influenced by a number of spatial and temporal factors such as habitat or the time of day an area is surveyed [8]. Understanding sources of variation in detection probability is therefore essential.

Raptors are generally considered useful indicators of biodiversity [9,10] and identifying their presence or absence can help deliver conservation outcomes [11,12]. However, they mostly occur at low densities [13,14] and this makes detecting and monitoring them difficult; it is not always possible to detect all the individuals of interest in a survey or monitoring programme, particularly where a species is rare or elusive. Developing estimates of detection probability is therefore a key part of such monitoring programmes, as is understanding the sources of variation in detection probability and how they can be managed.

Typical of many solitary-nesting savannah raptors, the white-headed vulture *Trigonoceps occipitalis* (Burchell 1824) occurs at low densities across its very large sub-Saharan range [15–17]. The species is critically endangered [18] and with a global population of approximately 5500 birds [19], they are difficult to find. To estimate the detection probability of large savannah raptors and the factors that affect it, in this study we used encounter rates of white-headed vultures during surveys in areas of known vulture occupancy. This aspect of our study meant that occupancy in our models was fixed at 1.0, which enabled the analysis to focus on detection probability itself by modelling variation in detection probability against a set of spatial and temporal covariates. Our objective was to determine how many times potential breeding areas of a large savannah raptor must be visited to assess presence or absence and the overall goal was to understand how the factors that affect detection probability should be incorporated into monitoring schemes for large and conspicuous species that occur at low densities.

## 2. Material and methods

### 2.1. Study sites

The study was conducted in two areas of Kruger National Park (KNP), in northeast South Africa. The southern area of approximately 3000 km^2^ and centred at S25.14460 E31.83620 is of low to moderate relief and vegetation consists mainly of mixed *Combretum* bush savannah with *Acacia nigrescens* and *Sclerocraya birrea* tree savannah [20]. The northern area of approximately 3300 km^2^ and centred on S23.16436 E31.42105 is flat to low relief. Vegetation in the northern area consists mainly of *Colophospermum mopane* open shrub savannah interspersed with more complex vegetation communities in riparian zones along the four main rivers in the study area (Bububu, Phugwane, Mphongolo and Shingwedzi).

Breeding sites of white-headed vultures were located using a combination of searches by vehicle and on foot, aerial surveys and information from park field rangers. Like most solitary-nesting raptors, white-headed vultures are generally considered to be territorial [15,21,22] and for the purposes of data collection and analysis, a territory was defined as a roughly circular area of 100 km^2^ centred on a known and active nest site. Territory size was a major assumption and was determined as a function of half the mean nearest neighbour distance of white-headed vultures in the study area [22]; this measure to estimate territory size has been used for other solitary-nesting vultures elsewhere [23]. Home ranges or territories are unlikely to be circular in reality, but very little is known about the territory or home range size of white-headed vultures. The 100 km^2^ size is similar to existing estimates [24,25] and the observed movements of individual adult vultures [26]. The assumption that white-headed vultures are territorial is also based on the fact that they nest solitarily and, usually, at regularly spaced distances from each other [17].

Visits to white-headed vulture territories were completed over three consecutive breeding seasons (2009–2011) and all by the same experienced observer (C.M.). Multi-season data were only collected in territories where nesting vultures used the same breeding site each year. During each visit to a territory, the entry and exit times were recorded and a minimum lag time of 30 min between visits to a territory was employed to ensure independence of survey samples. Thirty minutes was considered adequate because during this length of time a soaring raptor can theoretically cover the 10 or 12 km across the territory at a leisurely pace of 20 km h^−1^. Each territory was treated as spatially independent and it was assumed that the presence of another breeding territory adjacent to the one being surveyed did not affect the encounter rate. During each territory visit, we recorded the number, age and sex of any white-headed vultures that were encountered as well as their location and the length of time they were in view. False positives were avoided by recording confirmed observations only (i.e. clearly identified as white-headed vulture).

In other detection probability studies, the number of times each survey site is visited is often low and may average between four and five visits [27,28]. Based on this, the minimum number of visits was set to five and the analysis only included breeding territories that remained active throughout the breeding season (the closure assumption, see below).

### 2.2. Data analysis

Known breeding territories were treated as sample sites and each survey as an independent sample. From these data, estimates of detection probability were obtained using the occupancy model developed by MacKenzie *et al*. [4], expanded by MacKenzie *et al*. [29] and implemented in the programme Presence [30]. The model assumes that the population is closed, referred to as the closure assumption. By this, it is meant that there are no changes in occupancy (*ψ*) (i.e. colonizations or extinctions) at surveyed sites during the survey period; by using confirmed breeding territories that remained active throughout each breeding season, our study satisfied this assumption. Breeding territories that changed status during any year (e.g. a failed breeding attempt) were not included. Other assumptions of the model include independence of observations and no false detections, which were also met (see above).

### 2.3. Model selection

To identify sources of variation in detection probability, we selected nine variables in two categories (table 1) considered likely to have an effect on the probability of detecting a vulture and used them as covariates to create a set of 29 candidate models. We combined both site-specific and sample-specific covariates in the set of candidate models. Site-specific covariates do not vary within a season and are fixed and were also assumed to remain constant at each site between breeding seasons provided the birds nested in the same tree. Conversely, sample-specific covariates change with each survey of a site or during the survey period. Each covariate was used in the same number of models and all covariates were included in the global model. In each model, occupancy was fixed to 1.0 and the logit link was used to model detection probability against the different combinations of covariates [4].

**Table 1.** Variables used in candidate models to assess variation in detection probability during surveys of white-headed vultures in Kruger National Park, South Africa.

variable type, site-specific	
topography index (topog)	Number of 20 m contour intervals crossing two 1 000 m lines running north/south and east/west in the survey area, obtained from 1 : 50 000 topographic maps. High = more than five 20 m contours, low = five or fewer 20 m contour lines
habitat index (habitat)	Based on classifications for KNP occurring within each territory, in order of increasing vegetation density and multiplied by the topography index: 1 = shrub savannah; 2 = shrub and sparse tree savannah; 3 = tree savannah; 4 = open woodland; 5 = woodland
variable type, sample-specific	
time of year (date)	breeding season stage: (1) early (pre-egg laying, March to May); (2) mid: incubation and brooding (June to September); (3) late: larger pre-fledging chicks (October to December).
time of day (time)	time of entry to a survey area: <9.00; 9.00–12.00; 12.00–15.00; >15.00
time in territory (duration)	hours : minutes
mode of travel (mode)	(1) vehicle only;(2) vehicle and foot
time on foot (foot)	hours : minutes
average speed for visit (speed)	kilometres per hour
distance (distance)	(km) kilometres travelled inside territory

We used Akaike's information criterion (AIC) adjusted for small sample sizes (AICc) to rank all the candidate models and calculate their Akaike weights [31]. Once AICc for each model was calculated and the weights determined, a 95% confidence set of models was selected by starting with the highest weight model and then adding the weight of subsequently ranked models until the summed Akaike weights exceeded 0.95 [31]. Within this 95% confidence set, the relative importance of each covariate was assessed by summing the Akaike weight of each model in which it was included (∑CV_95_). To assess the importance of all the covariates, we summed the weight of all the models in which they were included across all the candidate models (∑CV_100_). Within the highest weight models, we used analysis of deviance (ANODEV) to determine the significance of each covariate and the amount of variance it explained.

The average detection probability estimated by the top model was used to calculate the number of times an area must be visited to determine the absence of white-headed vultures within a specified degree of confidence [3,32]. If the probability *F* of finding a species with a detection probability *p* after *N* visits is

2.1 $$F=1-{\left(1-p\right)}^{N},$$;

then the probability of not finding the species is (1 – *p*)*^N^*. Therefore in order to be 95% confident that the species is absent from a site (*F* = 0.95), the minimum number of visits required (*N*_min_) will be: *N*_min_ = log (1 – *F*)/log (1 – *p*).

## 3. Results

Data were collected from 359 visits to 17 confirmed white-headed vulture breeding territories between May 2009 and December 2011. The visits totalled 5481 km in the sample areas and a total of 102 birds was seen on 72 separate occasions. The mean sighting rate during territory visits was 1.87 birds per 100 km, which is approximately double the sighting rate of 0.92 birds per 100 km across Kruger in general (C. Murn, G. J. Holloway 2008–2013, unpublished data). The average number of visits to a breeding territory was 21 (range 5–47). White-headed vultures were detected at least once during the surveys in all except one of the breeding territories, and this site also had the fewest number of visits (5). The average number of visits per sighting was 5.5 (range 2–13). Table 2 shows a summary of the data obtained from territory visits.

**Table 2.** Summary data from visits to white-headed vulture breeding territories in Kruger National Park.

	total	mean per territory (range)
territory visits (*n* = 17)	359	21 (5–47)
distance (km)	5481	15.3 (3–51)
duration (h)	603	1 h 41 min (8 min to 9 h 19 min)
time on foot (h)	308	52 min (40 min to 7 h 10 min)
average speed (km h^−1^)	—	18.2 (2.7–55)

### 3.1. Covariate effects on detection probability

Four of the 29 candidate models contained over 95% of the AICc weight. The top model alone acquired 68.8% of the AICc weight and contained date, time, duration, mode and average speed. The second highest model acquired approximately 16% and contained the additional covariates of foot and distance (table 3).

**Table 3.** Twelve candidate models used to estimate detection probability of white-headed vultures during visits to occupied breeding territories. (Covariates affecting detection probability (*p*) were modelled with a constant occupancy parameter, *ψ*(.), and are listed under ‘model’. The models are ranked in descending order of support, according to their Akaike weight. The 95% confidence set of models, which accrued more than 95% of the Akaike weight, are shown in bold. Models 13–29 of the candidate set had no support and are not shown, as Akaike weights sum to 1. The p(global) model contains all covariates.)

model	AICc^a^	ΔAICc^b^	AICc wgt^c^	*K^d^*
***ψ*(.),p(date + time + duration + mode + speed)**	**293**.**30**	**0**.**00**	**0**.**6884**	**6**
***ψ*(.),p(date + time + duration + mode + foot + speed + distance**	**296**.**17**	**2**.**87**	**0**.**1639**	**8**
***ψ*(.),p(date + speed + duration)**	**297**.**67**	**4**.**37**	**0**.**0774**	**4**
***ψ*(.),p(topog + date + duration + speed)**	**299**.**67**	**6**.**37**	**0**.**0285**	**5**
*ψ*(.),p(global)	300.02	6.72	0.0239	10
*ψ*(.),p(topog + habitat + date + duration + speed)	301.58	8.28	0.011	6
*ψ*(.),p(mode + foot + speed + distance)	304.98	11.68	0.002	5
*ψ*(.),p(time + speed + distance)	305.29	11.99	0.0017	4
*ψ*(.),p(date + speed + distance)	305.88	12.58	0.0013	4
*ψ*(.),p(topog + foot + speed + distance)	307.01	13.71	0.0007	5
*ψ*(.),p(habitat + time + foot + speed)	307.36	14.06	0.0006	5
*ψ*(.),p(topog + habitat + time + speed)	307.76	14.46	0.0005	5

a Akaike's information criteria adjusted for small sample sizes.

b ΔAICc the difference between the model with the lowest AICc and that candidate model.

c Akaike weight for the candidate model.

d Number of parameters in the model.

The model ranked last in the 95% confidence set contained the topographic index covariate, while the habitat covariate did not appear in any of the higher-ranked models. Contributions of the different covariates to the top models are shown by their relative weights (table 4).

**Table 4.** Summed covariate weights from the 95% confidence set of models (∑Cw_95_) and all candidate models (∑Cw_100_) used to estimate sources of variation in detection probability of white-headed vultures in occupied territories. (Covariates with higher summed weights contribute more to the models that explain variation in detection probability.)

covariate	∑Cw_95_^a^	∑Cw_100_^b^
average speed	0.9582	0.9760
date	0.9582	0.9705
duration	0.9582	0.9692
entry time	0.8523	0.8551
travel mode	0.8523	0.8543
distance travelled	0.1639	0.1696
time on foot	0.1639	0.1672
topography	0.0285	0.0407
habitat	0	0.0121

a Summed weight of the covariate in the 95% confidence set of models.

b Summed weight of the covariate across all candidate models.

**Figure 1.**
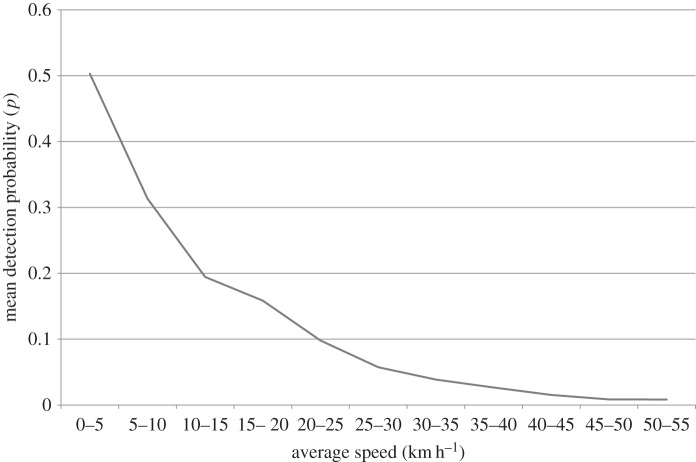
The effect of increasing average speed during a survey on the probability of detecting white-headed vultures in occupied breeding territories. Averaged detection probability and accompanying covariates from the highest ranked model were used (see the text for details).

Average speed during a survey was the most important covariate that affected detection probability. This covariate accumulated the highest relative weight (0.9760) across all the candidate models and was one of the top three covariates in the 95% confidence set of models. ANODEV showed that average speed was a significant covariate (p≪0.05) in every model and in the top model accounted for 6.3% of variance. Detection probability decreased as average speed during a territory visit increased (figure 1).

**Figure 2.**
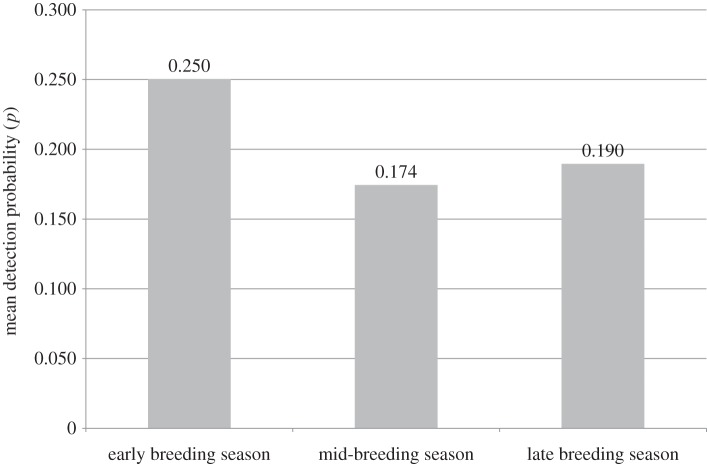
Mean detection probability of white-headed vultures in occupied breeding territories at different times of the year. Averaged detection probability and accompanying covariates from the highest ranked model were used (see the text for details). Early breeding season (March to May, pre-laying), mid-breeding season (June to September, incubation and brooding), late breeding season (October to December, larger chick and pre-fledging period).

Mode of travel was a significant covariate (p≪0.05) in the top two models and accounted for only 2.2% of variance. It was a significant covariate in all of the weighted models (table 3) in which it occurred. Duration (time spent in territory) was one of the top three covariates in the 95% confidence set but was a significant covariate only in the top model. Duration was correlated (0.754) with distance travelled and highly correlated (0.907) with time spent on foot—both of which were low-weighted covariates—and the interaction between these covariates may explain the lack of significance of duration in the other three models in the 95% confidence set. Time of year (date) occurred as a covariate in all the top models, but was not a significant covariate in any of them, possibly because there were too few data distributed across the range of values. Although detection probability varied throughout the year, it appears to be higher across all territories during the March to May period, before breeding pairs start to lay their eggs (figure 2).

Apart from not contributing to any of the models in the 95% confidence set and having very low Akaike weights, there was little influence on detection probability from the site-specific covariates of habitat and topography. The three covariates of average speed, time in territory (duration) and mode of travel all appeared as significant parameters in the top-weighted model (table 3), which suggests they are likely to provide high separate contributions to the higher-ranked models.

### 3.2. Number of visits to determine presence/absence

**Figure 3.**
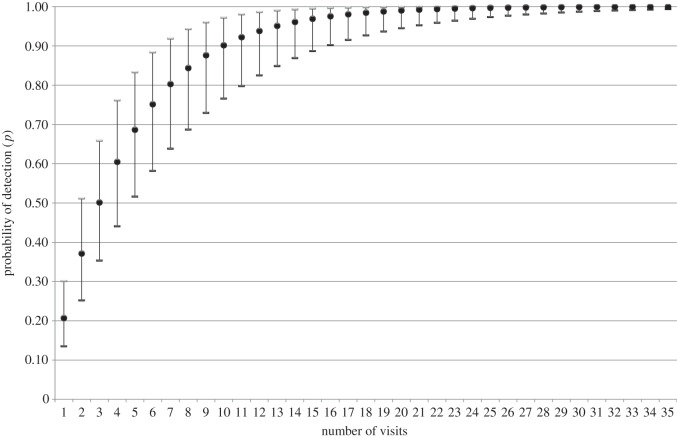
Cumulative probability of detecting a white-headed vulture in an occupied breeding territory after *N* repeated visits. Averaged detection probability and accompanying covariates from the highest ranked model were used (see the text for details). Vertical bars represent upper and lower 95% CIs.

Using the average detection probability (0.207) from the top model (table 3), the number of times a breeding territory needs to be visited to establish the absence of a white-headed vulture can be estimated. Figure 3 shows the probability of detecting a white-headed vulture at least once during increasing numbers of visits to a breeding territory using averaged values for the covariates in the top model (table 3). The number of visits before there is a 99% chance of establishing true absence is 21 (95% CI: 15–31). The mean number of visits required to be 95% confident that white-headed vultures are absent from a site is 13 (95% CI: 9–20).

## 4. Discussion

Recording white-headed vultures on only 72 occasions during more than 350 visits to confirmed breeding areas emphasizes that, even for a large and conspicuous territorial raptor, white-headed vultures are difficult to detect and monitor. Our results quantify this level of difficulty and by incorporating known occupancy into the modelling procedure to estimate detection probability, we have highlighted the extent to which repeated visits are necessary to detect large savannah raptors in their breeding territories and the importance of observer speed during surveys. Although averaged estimates from the top-weighted model were used, our purpose was not to define a fixed detection probability but to identify the variables that have the greatest influence on it. The covariates we identified can be applied to the development of monitoring protocols for large raptors that occur at low densities.

A major benefit of our study is that occupancy was known and this enabled attention to be focused on detection probability. Knowledge of occupancy is essential to correctly interpret the results of surveys with non-detection [33], because detection probability itself is not enough to distinguish between the probability of presence given non-detection (Pr = A|B) and the probability of detection given presence (Pr = B|A), which is not the same thing; confusing the two is known as ‘inverse fallacy’ [34]. To improve estimates of occupancy, variation in detection probability needs to be ‘controlled’ either before or after surveys [35], and while imperfect detection (i.e. *p* < 1) itself and covariates such as habitat and species abundance [36] cannot be controlled, other covariates, particularly temporal ones, can be. Our study showed these temporal covariates are important for determining detection probability and whether these are controlled before sampling via survey design or afterwards through modelling [37] is down to the objectives of the study [35] and the spatial pattern of species occurrence [7].

Highlighting average observer speed during territory visits as the most important covariate in our models seems an obvious result, but more intuitive covariates such as habitat, topography and distance travelled in the territory had little influence. If speed during a survey visit declines, the encounter probability increases dramatically. In practical terms, this means that surveys can be completed faster if desired, but each area would have to be visited many more times to have the same probability of encountering vultures as travelling slowly; figure 1 highlights that doubling survey speed from 10–15 km h^−1^ to 25–30 km h^−1^ results in an estimated decrease in detection probability of approximately 70%—from an already low probability of *p* = 0.1194 to an extremely low probability of *p* = 0.057.

Despite the two site-specific covariates of topography and habitat having little or no influence on detection probability in our study, it is still reasonable to expect that detection will vary in response to topography and habitat in other studies or areas. The interactive effects of vegetation combined with distance from the line transect were of major importance for the detection of large Serengeti mammals [38], although survey speed was maintained at less than 10 km h^−1^. This highlights the control of temporal covariates that affect detection probability via survey design, particularly where site-specific covariates are known to exert an influence. Likely explanations for topography and habitat having little influence in our study are that none of the breeding territories had particularly high relief and there were no habitat types that severely restricted visibility (e.g. closed woodland). It is possible that with average speed controlled for each territory visit, the effects of topography and habitat may have become more apparent.

The general trend of lower detection probability during the mid to late breeding season is attributable to one adult of a breeding pair attending the nest for incubation and rearing duties; during this time, the birds are less visible compared with when they are flying and this is likely to be the case for most large raptors that have long breeding seasons. This trend is the opposite of cliff-nesting Egyptian vultures *Neophron percnopterus* (Linnaeus 1758), which showed increasing probabilities of detection as the breeding season progressed [23]. However, the Egyptian vultures were monitored into the fledging period when more birds will be visible (and therefore detected) and it is likely that this feature of the survey design increased rates of detection later in the breeding season. For many survey programmes, determining occupancy at the beginning of a breeding season will be more useful than at other times because unoccupied areas can be removed from the survey schedule for the rest of the season. In our example, from a cost-benefit perspective, surveying white-headed vultures early in the season (i.e. before egg laying) is most effective because detection probability is higher and this maximizes the probability of detecting vultures in an area if they are in fact present. Concurrently, determining the absence of birds to within a degree of confidence is more likely to be achieved at this time. It is therefore clear that while increased survey effort (i.e. number of visits) for white-headed vultures early in the breeding season has a higher probability of determining occupancy (if not an actual breeding attempt), incorporating temporal factors into survey design and expectations is of fundamental importance.

With this in mind, it is likely that case-specific measures of detection probability need to be developed and applied [27], particularly where there is variation in breeding habitat but also where there are known differences in densities [36] or patterns of occurrence [7] or behaviour [39]. The use of *a priori* knowledge is essential for effective survey design [35], particularly for some species where sampling itself can violate the closure assumption through disturbance [40].

## 5. Conclusion

Detection probability is fundamental to occupancy studies and incorporating imperfect detection into survey frameworks will enhance site-occupancy and, by extension, species distribution models [41]. Surveys that *a priori* incorporate spatial and temporal factors that are grounded in background knowledge of the species will minimize cost outlays and the number of replicate surveys required. Survey methodologies that incorporate temporal covariates are applicable for two species of vultures and are likely to be for other large, solitary-nesting raptors. Variation in detection probability can be high and the results here have shown that surveys need to incorporate a range factors relevant to the detection of large savannah raptors such as speed of travel, date of survey and time spent in breeding territories.

## Data accessibility

The dataset supporting this study has been uploaded to Dryad Digital Repository: http://dx.doi.org/10.5061/dryad.72j20 [42].
